# Characterization of an attenuated SARS-CoV-2 variant with a deletion at the S1/S2 junction of the spike protein

**DOI:** 10.1038/s41467-021-23166-0

**Published:** 2021-05-13

**Authors:** Pui Wang, Siu-Ying Lau, Shaofeng Deng, Pin Chen, Bobo Wing-Yee Mok, Anna Jinxia Zhang, Andrew Chak-Yiu Lee, Kwok-Hung Chan, Rachel Chun-Yee Tam, Haoran Xu, Runhong Zhou, Wenjun Song, Li Liu, Kelvin Kai-Wang To, Jasper Fuk-Woo Chan, Zhiwei Chen, Kwok-Yung Yuen, Honglin Chen

**Affiliations:** 1 Department of Microbiology and State Key Laboratory for Emerging Infectious Diseases, Li Ka Shing Faculty of Medicine, The University of Hong Kong, Hong Kong, SAR China; 2 State Key Laboratory of Respiratory Disease, Institute of Integration of Traditional and Western Medicine, The First Affiliated Hospital of Guangzhou Medical University, Guangzhou Medical University, Guangzhou, China

**Keywords:** Viral infection, Live attenuated vaccines, Live attenuated vaccines, SARS-CoV-2

## Abstract

SARS-CoV-2 is of zoonotic origin and contains a PRRA polybasic cleavage motif which is considered critical for efficient infection and transmission in humans. We previously reported on a panel of attenuated SARS-CoV-2 variants with deletions at the S1/S2 junction of the spike protein. Here, we characterize pathogenicity, immunogenicity, and protective ability of a further cell-adapted SARS-CoV-2 variant, Ca-DelMut, in in vitro and in vivo systems. Ca-DelMut replicates more efficiently than wild type or parental virus in Vero E6 cells, but causes no apparent disease in hamsters, despite replicating in respiratory tissues. Unlike wild type virus, Ca-DelMut causes no obvious pathological changes and does not induce elevation of proinflammatory cytokines, but still triggers a strong neutralizing antibody and T cell response in hamsters and mice. Ca-DelMut immunized hamsters challenged with wild type SARS-CoV-2 are fully protected, with little sign of virus replication in the upper or lower respiratory tract, demonstrating sterilizing immunity.

## Abstract (web summary)

The S1/S2 junction of the SARS-CoV-2 Spike protein is emerging as a key factor in virulence and pathogenesis. Here, the authors characterise an attenuated strain of SARS-CoV-2 with deletions in the critical S1/S2 junction and observe enhanced replication, generation of potent adaptive immunity but reduced immunopathology in a hamster model of infection.

## Introduction

The emergence of SARS-CoV-2, a novel zoonotic origin β-coronavirus, has led to the first documented pandemic caused by a coronavirus^1,2^. The virus continues to circulate globally in humans with rapidly increasing numbers of infections and casualties each day (https://coronavirus.jhu.edu/map.html). While coronaviruses from bats and pangolins have been found to be closely related to SARS-CoV-2^3–6^, the direct ancestral virus which attained cross-species transmission and the intermediate animal host source of human infections have not been defined. In the past two decades, three coronaviruses have jumped the species barrier to infect humans^7^. SARS-CoV and MERS-CoV show limited human-to-human transmission ability while causing severe disease and mortality. In contrast, SARS-CoV-2, which uses the same human ACE2 binding receptor as SARS-CoV^6^, is highly transmissible and causes variable severity of disease, from asymptomatic infections to severe and fatal outcomes. Analysis of the SARS-CoV-2 genome reveals a distinct PRRA polybasic cleavage motif at the S1/S2 junction of the spike protein when compared to the most proximal animal coronaviruses yet detected^8^. Cleavage of spike into S1 and S2 subunits by the cellular protease furin promotes cell fusion mediated by S2, which is critical for cellular entry of coronavirus^9,10^. Polybasic cleavage sites are also found in some human coronaviruses, such as HKU1 (RRKRR) and OC43 (RRSR)^11,12^. While the role of these basic cleavage sites in human coronavirus infections is not clear, in avian influenza virus, acquisition of a polybasic cleavage motif facilitates infection of an increased variety of cell types^13–15^. However, the polybasic cleavage site is unlikely to be solely responsible for pathogenicity, as the 2003 SARS-CoV contains no such motif but was fatal in 10% of infected cases^16^. The polybasic cleavage site in SARS-CoV-2 is essential for infection of human lung cells^17^. It is speculated that acquisition of the PRRA polybasic motif in the spike protein of SARS-CoV-2 provides the virus with its unique cross-species transmissibility; this motif therefore appears to serve as a pathogenic element in human infections^10,17^. If the PRRA polybasic cleavage motif was not a natural functional component in the original virus and acquired after cross-species transmission, it is postulated that it may not be stable during infection in its new hosts or may need further adaptation. Indeed, a panel of SARS-CoV-2 variants with various lengths of deletion spanning the PRRA polybasic cleavage motif at the spike protein S1/S2 junction were identified in cultured cells and at low levels in clinical specimens, suggesting the S1/S2 junction may be under selection pressure as the SARS-CoV-2 virus circulates in humans^18,19^. Initial characterization revealed that deletion at the S1/S2 junction causes SARS-CoV-2 virus attenuation in hamsters and prompted a further study to understand the properties of these deletion mutants in in vitro and in vivo systems. It remains to be determined whether some of these deletion variants may become more prevalent in humans as SARS-CoV-2 circulation continues. On the other hand, these attenuated SARS-CoV-2 variants may hold promise as candidate live vaccines and it is important that their potential in this regard be evaluated.

To understand the role of the polybasic cleavage site in infection and replication of SARS-CoV-2 and determine the stability of deletion variants, a previously characterized deletion mutant^18^, Del-Mut-1, was further adapted in Vero E6 cells to obtain a highly attenuated variant of SARS-CoV-2 virus, designated Ca-DelMut (cell-adapted deletion mutant). We analyzed the growth properties of this variant in cells and evaluated its pathogenicity in a hamster model. While it is attenuated in the ability to cause disease in animals, Ca-DelMut replicates to a higher titer in Vero E6 cells than a wild-type (WT) SARS-CoV-2 virus. High titers of neutralizing antibodies and significant numbers of virus-specific CD4^+^ and CD8^+^ T cells were detected in animals previously immunized with the Ca-DelMut variant. However, infection with Ca-DelMut does not induce the high levels of proinflammatory cytokines seen in WT virus infections. Importantly, Ca-DelMut-immunized hamsters showed full protection against challenge with two different strains of WT SARS-CoV-2 virus.

## Results

### Growth and pathogenic properties of Ca-DelMut in vitro and in vivo

**Fig. 1:**
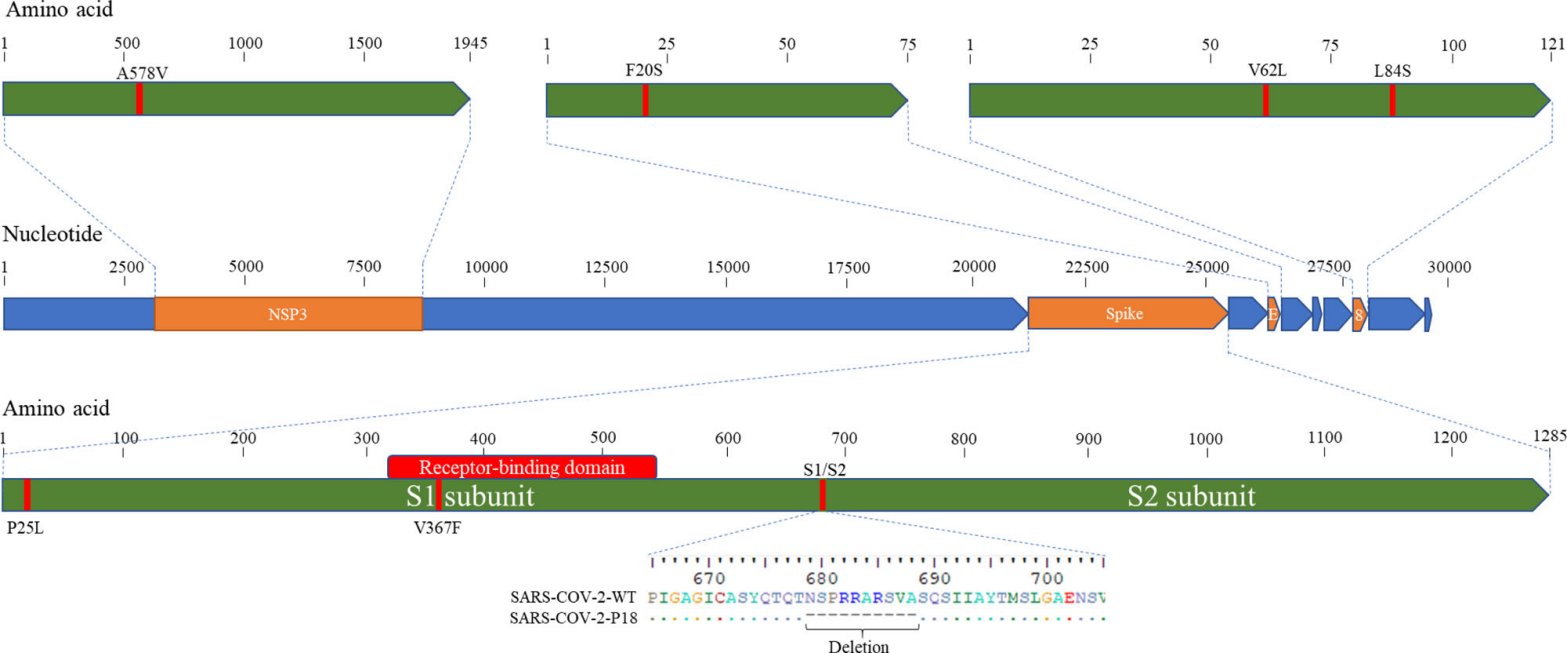
Schematic diagram of the SARS-CoV-2 genome showing the deletion and mutations of Ca-DelMut. Del-Mut-1 virus^18^ was serially passaged in Vero E6 cells at 33 °C (10 passages) and 30 °C (8 passages). Virus from the 18th passage was designated as Ca-DelMut live attenuated SARS-CoV-2 virus and amplified to prepare a virus stock. Ca-DelMut was sequenced by the Sanger method; mutations are shown in the diagram and in Supplementary Table 1.

**Fig. 2:**
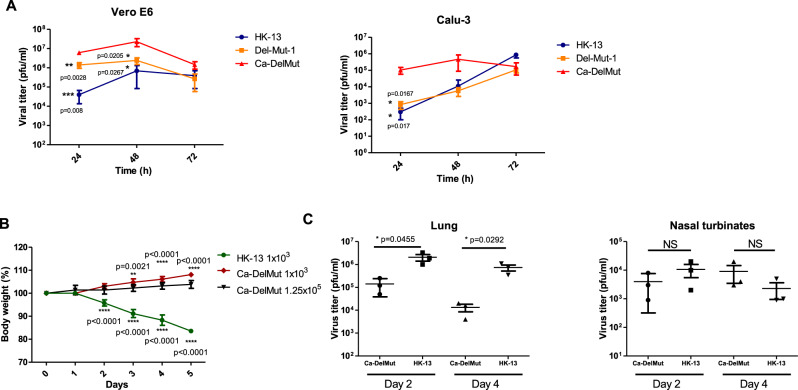
Replication efficiency of Ca-DelMut in vitro and in vivo. **A** Vero E6 or Calu-3 cells were infected with Ca-DelMut and other viruses at 0.01 MOI and cultured at 37 °C. At the indicated time points, supernatants were collected, and virus titer determined by plaque assay in Vero E6 cells. For each time points, 3 biological replicates were performed. Error bars represent mean ± SD (*n* = 3). Statistical comparisons between means (*n* = 3) were performed by one-way ANOVA with Tukey post-test: ^***^p < 0.001, ^**^p < 0.01, ^*^p < 0.05. **B** Ca-DelMut infection in hamsters. Hamsters were infected intranasally with either Ca-DelMut (*n* = 9) or wild-type (WT) (*n* = 9) viruses at different doses, as indicated. Body weight was monitored for 5 days. Error bars represent mean ± SD (*n* = 3). Statistical comparisons between means were performed by two-way ANOVA with Bonferroni post-test: ^***^p < 0.001, ^**^p < 0.01. **C** Replication of Ca-DelMut in lung and nasal turbinate tissues. After virus challenge (1 × 10^3^ pfu), lung and nasal turbinate tissues were collected from hamsters at days 2 and 4, then homogenized and virus titer determined. Error bars represent mean ± SD (*n* = 3). Statistical comparisons between means were performed by one-way ANOVA with Tukey post-test: ^**^p < 0.01, ^*^p < 0.05, NS: not significant.

We previously identified and characterized a panel of SARS-CoV-2 variants containing 15–30 bp deletions at the S1/S2 junction of the spike protein. One of the variants, Del-Mut-1, which contains a 30 bp (10 amino acid) in-frame deletion spanning the PRRA polybasic cleavage motif was shown to be attenuated in a hamster infection model^18^. We further passaged this mutant in Vero E6 cells and obtained a variant with additional mutations in multiple genes in the background of Del-Mut-1, designated Ca-DelMut (Fig. 1 and Supplementary Table 1). Growth properties of Ca-DelMut, parental Del-Mut-1, and a WT SARS-CoV-2 (HK-13) were analyzed in Vero E6 and Calu-3 cells. It is interesting to note that in Vero E6 cells both Del-Mut-1 and Ca-DelMut grow to a significantly higher titer at the 24 and 48 h time points than the WT virus, with Ca-DelMut showing the strongest growth ability in both cell types (Fig. 2A). Notably, Ca-DelMut also replicated faster at 30 °C than the WT virus (HK-13) (Supplementary Fig. 1). Using HIV pseudoviruses (expressing WT or mutant spike proteins) to infect 293T-hACE2 cells, it was found that deletion of 30 bp in the S1/S2 junction significantly enhances cell entry (Supplementary Fig. 3C). Besides the 30 bp deletion, we also found that a mutation in the ORF8 (L84S) of Ca-DelMut increases protein expression, but it is not known if this mutation contributes to virus growth ability (Supplementary Fig. 3A). Biological significance of substitutions in NSP3a (A578V) and E (F20S) is not clear.

Attenuation of Del-Mut-1 was reported previously^18^. To further characterize the Ca-DelMut variant of SARS-CoV-2, we infected groups of hamsters with variant or WT SARS-CoV-2 strains. While inoculation with 10^3^ plaque forming units (pfu) of HK-13 (*n* = 9) caused significant body weight loss post-infection, no apparent body weight loss was observed in Ca-DelMut (1.25 × 10^5^ (*n* = 6) and 10^3^ (*n* = 9) pfu)-infected hamsters, although more modest bodyweight gain was observed in the high-dose group, which may suggest a residual pathogenic effect associated with the higher inoculum (Fig. 2B). Histopathological analysis showed only mild regional alveolar septal infiltration and blood vessel congestion, with no obvious bronchiolar epithelium desquamation or luminal debris, no alveolar space infiltration or exudation, and no pathological changes in the intestines of Ca-DelMut-infected hamsters, which are markedly different from the severe pathology observed in WT-virus-infected animals (Supplementary Fig. 2). To understand the molecular basis of attenuation of Ca-DelMut, we tested spike protein cleavage efficiency in cells. Deletion of the furin-cleavage site abolished S1/S2 cleavage of spike protein in 293T cells while other mutations (P25L and V367F) did not affect cleavage (Supplementary Fig. 3B). Examination of virus replication in lung and nasal turbinate tissues showed that Ca-DelMut replicates actively in the nasal turbinate tissues but that replication efficiency is much lower than that of HK-13 in hamster lungs (Fig. 2C). Staining of viral nucleocapsid protein (NP) in the lungs of infected hamsters showed that Ca-DelMut tends to be restricted to pneumocytes, whereas WT virus can infect both epithelial cells and pneumocytes effectively (Supplementary Fig. 4). These results indicate that Ca-DelMut has altered tissue tropism and is more likely to infect and replicate in the upper respiratory tract.

### Immune response to infection with Ca-DelMut in hamsters

**Fig. 3:**
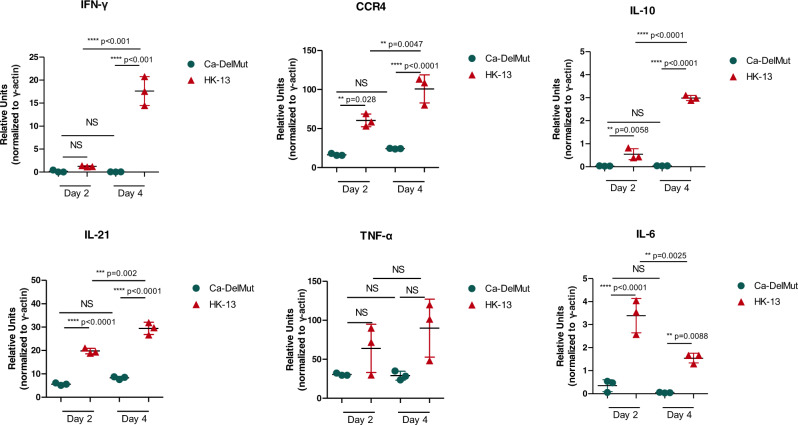
Proinflammatory cytokine response profiles in Ca-DelMut- and wild-type-virus-infected hamsters. Hamsters were infected intranasally with 1 × 10^3^ pfu of either Ca-DelMut (*n* = 9) or WT HK-13 virus (*n* = 9). At days 2 and 4, RNA was extracted from lung tissues of infected hamsters (*n* = 3) and cDNA synthesized using oligo dT primers. Expression of different proinflammatory cytokines was examined by qPCR; relative mRNA levels (relative units) are normalized to an internal reference gene (hamster γ-actin), and the comparative Ct (2-ΔΔCt) method utilized to calculate the cytokine expression profile. Statistical comparisons between means were performed by one-way ANOVA with Tukey post-test: ^****^p < 0.0001, ^***^p < 0.001, ^**^p < 0.01, ^*^p < 0.05, NS: not significant.

**Fig. 4:**
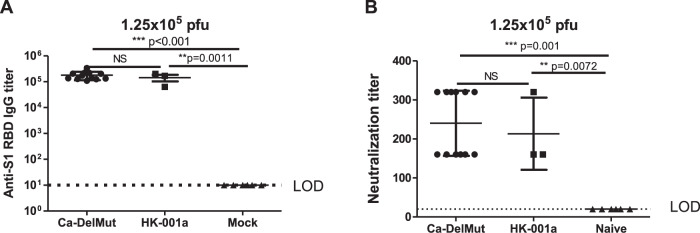
Antibodies induced by Ca-DelMut immunization of hamsters. Hamsters were immunized intranasally with 1.25 × 10^5^ pfu of either Ca-DelMut (*n* = 12) or wild-type virus (HK-001a) (*n* = 3)^18,25^, or mock immunized (*n* = 6). At day 21, blood was collected from hamsters and tested for (**A**) anti-S1 RBD-specific IgG titers and (**B**) neutralization activity against the HK-13 virus strain. Error bars represent mean ± SD. LOD: level of detection. Statistical comparisons between means were performed by Student’s *t*-test (2-tailed): ^*^p < 0.05, NS: not significant.

**Fig. 5:**
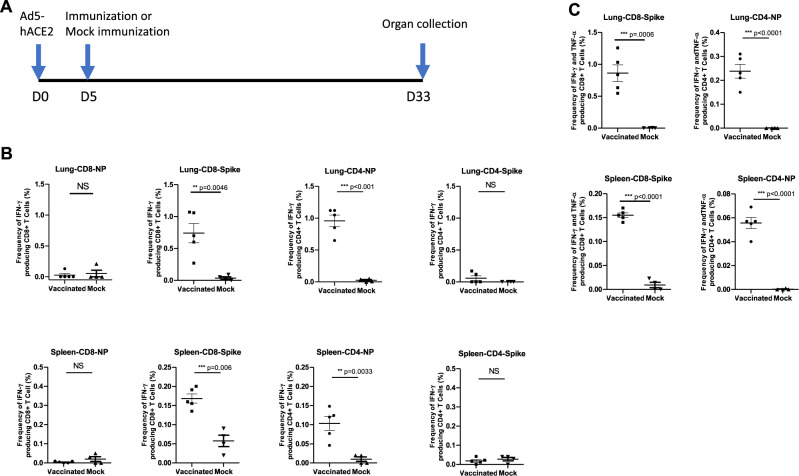
T cell response to Ca-DelMut immunization in Ad5-hACE2 transduced mice. **A** Timeline of Ad5-hACE2 transduction and immunization of Ad5-hACE2 transduced mice with Ca-DelMut. **B** Ca-DelMut induces IFN-γ positive CD4^+^ and CD8^+^ T cell responses in Ad5-hACE2 transduced mice. Four weeks after immunization, splenocytes and lung cells were obtained and stimulated with either spike or NP peptide pools or incubated without peptides overnight in the presence of BFA. Surface markers were stained, and cells fixed and permeabilized. Intracellular cytokines were then stained with antibodies. Sample data were acquired using a BD FACSAria III cell sorter. IFN-γ^+^ CD4^+^ and CD8T cells in immunized (*n* = 5) and naive groups (*n* = 4) were compared. Statistical comparisons between means were performed by Student’s *t*-test (2-tailed): ^****^p < 0.0001, ^***^p < 0.001, ^**^p < 0.01, NS: not significant. **C** Frequency of polyfunctional IFN-γ/TNF-α double positive CD4^+^ and CD8^+^ cells in Ca-DelMut virus immunized (*n* = 5) and naive mice (*n* = 4). Statistical comparisons between means were performed by Student’s *t*-test (2-tailed): ^****^p < 0.0001, ^***^p < 0.001, ^**^p < 0.01, NS: not significant.

Impaired or dysfunctional immune responses have been characterized as an important mechanism of pathogenesis in human SARS-CoV-2 infections^20–22^. To test if infection with Ca-DelMut may induce a different immune response from that elicited by WT SARS-CoV-2, we examined interferon and cytokine expression in the lung tissues of virus-infected hamsters. In contrast to infection with WT HK-13 strain, we found that the Ca-DelMut variant does not provoke elevated levels of cytokines in infected hamsters (Fig. 3 and Supplementary Fig. 5). Aberrant activation of IL-6 has been recognized as an important biomarker of disease severity in SARS-CoV-2-infected patients^22–24^. Remarkably, activation of IL-6 was only observed in HK-13-strain-infected hamsters but not in those infected with Ca-DelMut variant. We then analyzed the adaptive immune response in hamsters previously infected with Ca-DelMut or WT virus. Levels of receptor binding domain (RBD)-specific antibodies in sera collected 3 weeks after infection were determined. Sera from hamsters previously infected with Ca-DelMut or that had recovered from WT virus (HK-001a)^25^ infection showed significant induction of RBD-specific antibodies (Fig. 4A and Supplementary Fig. 6A). Cell-based neutralization assays also demonstrated strong neutralizing activity against the WT virus strain HK-13 in sera collected from both Ca-DelMut-variant- and WT SARS-CoV-2 virus (HK-13)-infected hamsters (Fig. 4B and Supplementary Fig. 6B). Effective T cell activation is critical to prevent severe disease in COVID-19 patients and strong T cell responses have been shown in convalescent individuals^26,27^. We examined whether immunization with Ca-DelMut induces a specific T cell response in animals. Because there is lack of reagents available for immunological analysis of hamsters, we utilized an Ad5-hACE2 adenovirus transduced mouse model to estimate T cell responses to Ca-DelMut immunization. Mice were transduced with Ad5-hACE2 virus through the nasal route 5 days prior to immunization with Ca-DelMut SARS-CoV-2 variant and T cell responses in lungs and spleens analyzed after 4 weeks (Fig. 5A). Using T cell epitopes specific to the NP and spike proteins of SARS-CoV-2, we found that immunization with Ca-DelMut induces significant levels of CD4^+^ and CD8^+^ T cells in both the lungs and spleens of mice (Fig. 5B, C). These results indicate that infection with Ca-DelMut leads to an altered immune response that does not induce the elevated levels of proinflammatory cytokines seen in WT SARS-CoV-2 virus infection. Nonetheless, Ca-DelMut elicits a strong adaptive immune response, as demonstrated by significant levels of neutralizing antibody production and induction of specific CD4^+^ and CD8^+^ T cells recognizing viral NP and spike proteins, respectively.

### Infection with Ca-DelMut confers full protection against SARS-CoV-2 infection with sterilizing immunity

**Fig. 6:**
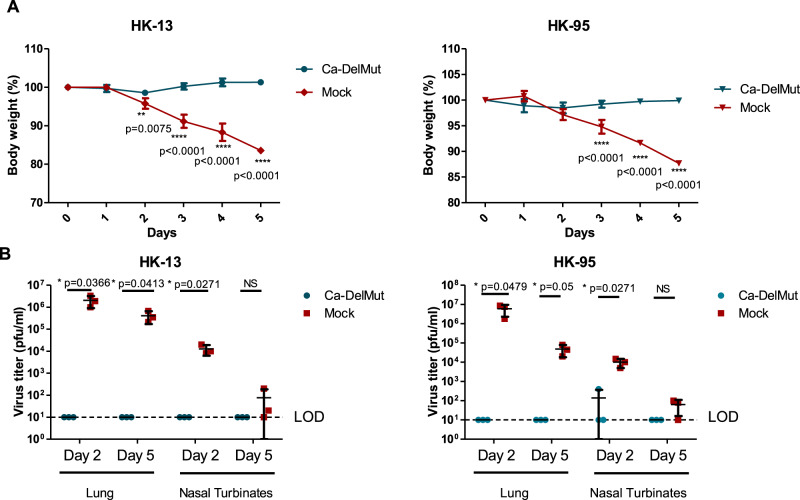
Ca-DelMut immunization protection against WT virus challenge in hamsters. Hamsters were inoculated with 1.25 × 10^5^ pfu Ca-DelMut (*n* = 12) or mock immunized (*n* = 12). At day 28 after immunization, hamsters were challenged with 1 × 10^3^ pfu of either HK-13 (*n* = 6) or HK-95 (*n* = 6) virus. **A** Body weight and disease symptoms were monitored for 5 days. Error bars represent mean ± SD (*n* = 3). Statistical comparisons between means were performed by two-way ANOVA with Bonferroni post-test: ^****^p < 0.0001, ^***^p < 0.001, ^**^p < 0.01. **B** At days 2 and 5 post-infection, lungs and nasal turbinate tissues were collected for virus titration and histopathological study. Error bars represent mean ± SD (*n* = 3). Statistical comparisons between means were performed by Student’s *t*-test (2-tailed): ^*^p < 0.05, NS: not significant.

**Fig. 7:**
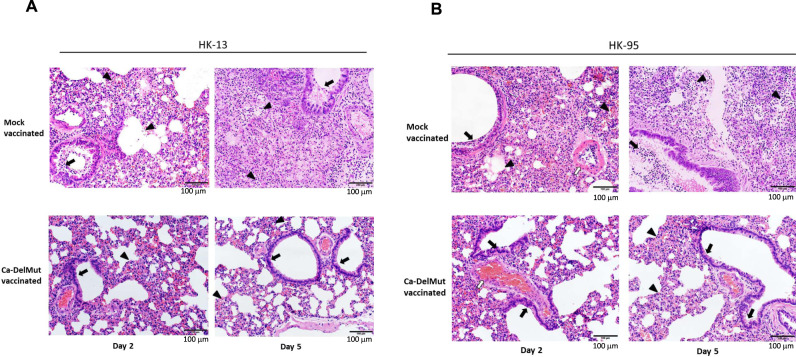
Histopathological analysis of lung pathology in WT-virus-challenged Ca-DelMut- and mock-immunized hamsters. At day 28 after immunization, hamsters were challenged with 1 × 10^3^ pfu of either HK-13 or HK-95 virus. At days 2 and 5 post-infection, lungs were collected, fixed, processed into paraffin blocks, and sections H&E stained. **A** Challenge with HK-13. Day 2: Mock-vaccinated hamster lungs showed bronchiolar epithelial cell death and the bronchiolar lumen filled with exudate and cell debris (arrow). Diffuse alveolar infiltration and focal hemorrhage were also seen (arrowheads). Ca-DelMut-vaccinated hamster lungs showed regional alveolar septal infiltration and blood vessel congestion (arrowhead), but no obvious bronchiolar epithelial cell death (arrow). Day 5: Lungs of mock-vaccinated hamsters showed severe alveolar infiltration and exudation (arrowheads), as well as bronchiolar luminal exudation (arrow). Vaccinated hamster lungs showed focal alveolar septal infiltration (arrowheads), while the bronchiolar epithelium appeared normal with no luminal secretion or cell debris (arrows). Images were representatives of three independent experiments. **B** Challenge with HK-95. Day 2: Mock-vaccinated hamster lungs showed bronchiolar epithelial cell death with luminal cell debris (arrow) and diffuse alveolar infiltration with focal hemorrhage and exudation (arrowheads), with a medium sized blood vessel showing severe endotheliitis (open arrow). Vaccinated hamster lungs showed regional alveolar septal infiltration and blood vessel congestion; a blood vessel appeared to be normal (open arrow), and no obvious bronchiolar epithelial cell death was observed (arrows). Day 5: Lungs of mock-vaccinated control hamsters showed severe alveolar infiltration and exudation (arrowheads) and bronchiolar luminal cell debris (arrow). Vaccinated hamster lungs showed focal alveolar septal infiltration (arrowheads), while the bronchiolar epithelium appeared normal without luminal secretion (arrows). Scale bar: 100 µm. Images were representatives of three independent experiments.

Because infection with Ca-DelMut causes no apparent disease in hamsters while inducing a strong neutralizing antibody response in hamsters and T cell activation in transduced mice expressing the human ACE2 receptor, we examined the potential of Ca-DelMut as a live attenuated virus vaccine to prevent SARS-CoV-2 virus infection and disease. Four weeks after infection with Ca-DelMut live attenuated virus, hamsters were challenged with WT SARS-CoV-2 viruses. Two strains of SARS-CoV-2, HK-13 and HK-95, were used in the challenge experiment. HK-95 contains a D614G substitution in the spike protein, which has been suggested to bestow SARS-CoV-2 with higher infectivity in humans^28^. Negligible body weight loss was observed in Ca-DelMut-vaccinated hamsters infected with either strain of WT virus, whereas control hamsters not immunized with Ca-DelMut lost about 12–15% of their body weight by day 5 post-infection (Fig. 6A and Supplementary Fig. 7). Analyses of virus replication in the lung and nasal turbinate tissues of re-challenged hamsters showed that Ca-DelMut infection provides sterilizing immunity against subsequent challenge with either HK-13 or HK-95 SARS-CoV-2, with the quantity of virus being either very low, as seen in one-third of the HK-95 challenge group on day 2, or below the level of detection in both lung and nasal tissues on days 2 and 5 post-infection (Fig. 6B and Supplementary Fig. 7). We found that HK-95 replicates more efficiently than HK-13, and it is possible that a higher inoculum of Ca-DelMut may be needed to ensure complete blockage of virus replication in the upper respiratory tract of HK-95-challenged hamsters. Histopathological analysis showed that mock-vaccinated hamster lungs collected at day 5 post-infection with either WT virus strain showed extensive alveolar exudation and infiltration; bronchiolar epithelial cell death with luminal exudation and cell debris were also observed (Fig. 7). In contrast, Ca-DelMut-inoculated hamsters challenged with either strain of WT SARS-CoV-2 virus only experienced mild regional alveolar septal infiltration and blood vessel congestion at days 2 and 5 post-infection. No other pulmonary histopathological changes were observed. Our experiment also showed that a lower inoculum of Ca-DelMut (1 × 10^3^ pfu) is sufficient to provide full protection against WT virus challenge (Supplementary Fig. 8). These data indicate that prior infection with live attenuated Ca-DelMut variant virus provides complete protection against infection with WT SARS-CoV-2 viruses in hamsters.

## Discussion

Coronaviruses are zoonotic pathogens with distinct cross-species transmissibility^29^. Besides 229E, OC43, HKU-1, and NL63, which have long been circulating in humans, SARS-CoV, MERS-CoV, and SARS-CoV-2 have jumped the species barrier to infect humans in recent years^7^. SARS-CoV has not been detected since 2004, while MERS-CoV is restricted to a few countries in the Middle East with only sporadic human transmissions since 2012^30^. SARS-CoV-2 virus utilizes the same cellular receptor as SARS-CoV-1, ACE2, for mediating human infection but has exhibited a distinctive infectivity and transmissibility profile since it was first recognized in humans in Wuhan, China, in December 2019^6^. There is strong interest in understanding how SARS-CoV-2 has acquired the unique ability to infect and transmit efficiently in humans. SARS-CoV-2 contains a PRRA polybasic motif not seen in the most closely related bat and pangolin coronaviruses currently known^3,5,8^. A peptide assay has shown that the polybasic cleavage site harbored within SARS-CoV-2 is easily accessible to proteases that activate the coronavirus spike protein^10^. Although furin-like polybasic cleavage sites have been found in the human coronaviruses OC43 and HKU1^12^, the presence of a PRRA motif at the S1/S2 junction of the spike protein of SARS-CoV-2 virus is considered a critical element for enhanced coronavirus infectivity in humans and zoonotic potential^17,31^. This contention is supported by structural comparison of SARS-CoV-2 and bat RaTG13 spike proteins, which revealed that acquisition of the furin-cleavage site could facilitate a more optimal confirmation for spike protein to bind to the ACE2 receptor^32^. How this polybasic cleavage motif was acquired by SARS-CoV-2 remains unclear. Since both bat RaTG13- and pangolin SARS-CoV-2-related viruses that contain highly similar spike protein receptor domains to SARS-CoV-2 do not contain such motifs^3,33^, it is possible that this polybasic cleavage site was acquired either through recombination with a human coronavirus containing the motif or through another mechanism during virus replication in the early stage of human transmission. If the polybasic cleavage site is one of the essential elements providing SARS-CoV-2 with increased infectivity and pathogenicity in humans, removal of this determinant could logically attenuate SARS-CoV-2 into a mild respiratory virus similar to the less pathogenic common coronaviruses currently circulating. Our previous report on a panel of attenuated variants with deletions at the S1/S2 junction supports this contention^18^. This study has obtained a further Vero E6-adapted variant, Ca-DelMut, with additional mutations in spike and other genes (Fig. 1). One of the mutations, V367F, is situated inside the RBD domain and further study is required to understand if it may associate with altered receptor binding activity. Characterization of Ca-DelMut reveals that it has low pathogenicity and does not provoke an inflammatory response in hamsters, and that it induces adaptive immunity protective against subsequent infection with more pathogenic SARS-CoV-2 strains. Ca-DelMut attenuated virus could also be a useful tool for studying SARS-CoV-2 replication, host tissue tropism, and transmissibility.

Although both SARS-CoV-1 and SARS-CoV-2 utilize ACE2 to mediate infection, the distinctive infectivity and pathogenicity displayed by SARS-CoV-2 is likely to be associated with the acquisition of a polybasic furin-cleavage site in the spike protein, which, together with enhanced binding affinity of the SARS-CoV-2 RBD for ACE2, would significantly broaden the tissue tropism of this virus^17,32^. Deregulated innate immunity during the early stage of infection in the upper respiratory tract may determine the subsequent outcome regarding dissemination to the lower respiratory tract and disease severity^20,34^. We found that Ca-DelMut replicates to comparable levels to WT virus in the nasal turbinates but less effectively than WT virus in the lungs (Fig. 2C). Importantly, while replication of Ca-DelMut variant was observed in lung tissues, the elevated expression of proinflammatory cytokines elicited in WT-virus-infected hamsters was not detected (Fig. 3 and Supplementary Fig. 5). These observations clearly suggest that the polybasic cleavage motif is a virulence element in SARS-CoV-2 and that its removal makes SARS-CoV-2 much less pathogenic, and more similar to a common cold respiratory coronavirus. We believe that SARS-CoV-2 will continue to undergo further adaptation as it circulates in humans. Our previous study revealed that variants with deletions at the S1/S2 junction are present at low levels in clinical specimens^19^. In 2003, the SARS-like coronavirus characterized from civet cats and early-outbreak human SARS-CoV isolates contained a 29 bp sequence in the ORF8 sequence, which was deleted following its subsequent circulation in humans^35^. Deletions in ORF7b and ORF8 have also been observed in SARS-CoV-2, although the significance of these alterations is currently unknown^36,37^. Interestingly, two ORF8 mutations, V62L and L84S, were identified in the Ca-DelMut variant (Fig. 1 and Supplementary Table 1). It remains to be seen if continued evolution of SARS-CoV-2 in humans will subsequently select for less-pathogenic variants similar to Ca-DelMut, or with other mutations. Further studies on the role of ORF8 in host adaptation are required.

Some early studies suggested that anti-SARS-CoV-2 antibodies decline rapidly, while recent studies suggest the presence of immunological memory in naturally infected individuals^38–40^. Several SARS-CoV-2 vaccines have been developed and are being used in humans^41–45^. However, it remains to be seen if current vaccine strategies will be able to provide adequate and sufficiently long-lasting immunity to prevent infection and alleviate disease severity and spread. This study showed that Ca-DelMut induces a different innate immune response to that observed in WT virus infections in an animal model and evokes sterilizing protective adaptive immunity against challenge with WT virus (Figs. 4–6). Because Ca-DelMut is attenuated, it does not provoke proinflammatory cytokines that could interfere with the induction of adaptive immunity. It is possible that the Ca-DelMut variant may be able to stimulate more balanced and long-lasting immunity; further study is required to comprehensively compare the immune profiles induced by Ca-DelMut and WT SARS-CoV-2 viruses. Understanding the immunological response to infection with Ca-DelMut may provide valuable information for improving current vaccine strategies. Given the high replication efficiency, low pathogenicity, and maintenance of original antigenicity of Ca-DelMut, it may be an ideal strain for production of an inactivated vaccine for use in humans^46^.

## Methods

### Generation of cell-adapted Del-Mut (Ca-DelMut) virus

Del-Mut viruses were prepared as previously described^18^. To generate Ca-DelMut virus, the Del-Mut-1 virus was serially passaged 10 times in Vero E6 (ATCC) cells at 33 °C and then passaged at 30 °C 8 times. For each passage, incubation time was 2–3 days, depending on the occurrence of cytopathic effects (CPEs). After the 18th passage, the virus was amplified in a T75 Flask, then titered by plaque assay. Viral RNA from the cell-adapted variant (Ca-DelMut) was amplified by RT-PCR and sequenced using the Sanger chain termination method. List of primers used in this study is provided in Supplementary Table 2.

### Growth kinetics

Confluent Vero E6 or Calu-3 (ATCC) cells were infected at 0.01 MOI (multiplicity of infection) with the indicated viruses and incubated for 3 days. Virus supernatant was collected at the indicated time points. Virus titers were determined by plaque assay using Vero E6 cells.

### Plaque assay

Confluent Vero E6 cells in 6-well format were incubated with 10-fold serially diluted virus for 1 h. After adsorption to Vero E6 cells, virus supernatant was discarded. The virus supernatant was then discarded and cells were washed and overlaid with 1% agarose in DMEM and incubated for 3 days at 37 °C. Cells were fixed with 10% formaldehyde for 1 day. Agarose gels were then removed, and plaques stained with 1% crystal violet and counted.

### Immunization and challenge of hamsters

Male and female Syrian hamsters, 6–10-week-old, were obtained from the Chinese University of Hong Kong Laboratory Animal Services Centre through The University of Hong Kong Centre for Comparative Medicine Research. Groups of 7–8-week-old golden Syrian hamsters were anesthetized intraperitoneally with ketamine and xylazine and then immunized intranasally with 1.25 × 10^5^ pfu of Ca-DelMut (*n* = 12 per group) or HK-001a (*n* = 6) virus or mock immunized with PBS (*n* = 6). Body weight and disease symptoms were monitored daily. At day 21, sera were collected from hamsters for anti-spike RBD IgG and neutralizing antibody determination. At day 28, Ca-DelMut- and mock-immunized hamsters were challenged with WT virus (HK-13 or HK-95) at a dose of 1 × 10^3^ pfu. Body weight and disease symptoms were monitored daily and at days 2 and 5, lung and nasal turbinate tissues were collected for histopathology and virus titration by plaque assay. For low-dose immunization, hamsters (three per group) were immunized with 10^3^ pfu of either Ca-DelMut or WT (HK-13) virus, or mock immunized with PBS. All experiments involving SARS-CoV-2 were conducted in a biosafety level 3 laboratory. All animal studies were approved by the Committee on the Use of Live Animals in Teaching and Research, The University of Hong Kong (CULATR 5359-20 for hamster experiment and CULATR 5350-20 for mouse experiment).

### Pathogenicity of Ca-DelMut and wild-type SARS-CoV-2 virus in hamsters

Hamsters were challenged intranasally with 1 × 10^3^ (*n* = 9) or 1.25 × 10^5^ (*n* = 6) pfu of Ca-DelMut or 1 × 10^3^ pfu of WT HK-13 virus (*n* = 9). Body weight and disease symptoms were monitored daily. At days 2 and 4, lung and nasal turbinate tissues were collected for histopathological study, determination of proinflammatory cytokine expression and virus titration.

### Neutralization assay

Heat-inactivated sera from Ca-DelMut-challenged hamsters were 2-fold serially diluted in DMEM medium and incubated with 100 pfu of the indicated virus at 37 °C for 1 h. The mix was added to confluent Vero E6 cells and incubated for 4 days at 37 °C. Naive and WT (HK-001a or HK-13) challenged sera were used as controls. After 4 days, CPE was detected by microscopy, with the neutralization endpoint being the highest serum dilution causing 50% inhibition of CPE.

### ELISA

A hamster anti-spike RBD IgG detection kit (Wantai-Bio) was used to detect RBD-specific antibodies. Procedures were conducted in accordance with the manual. Briefly, heat-inactivated sera from Ca-DelMut-challenged hamsters were 10-fold serially diluted and added to the plate and incubated at 37 °C for 30 min. Sera from mock- and WT (HK-001a or HK-13)-challenged hamsters were included as controls. The plate was washed 5 times and then incubated with horseradish peroxidase (HRP)-conjugated goat anti-hamster IgG secondary antibody (Abcam) at 37 °C for 30 min. After washing, color development solution (3,3′,5,5′-tetramethylbenzidine) was added and the plate incubated at 37 °C for 15 min. Stop solution (0.5 M sulfuric acid) was added and absorbance at 450 nm measured.

### Quantification of expression of proinflammatory cytokines and chemokines

Expression of proinflammatory cytokines was quantified using a qRT-PCR technique similar to that described in a previous study^47^. Briefly, total RNA was extracted from hamster lungs using RNAzol RT reagent (MRC) according to the manual. cDNA was synthesized using a High Capacity cDNA Reverse Transcription Kit (Invitrogen) and oligo dT primers following the protocol provided. qPCR was performed using SYBR Premix Ex Taq (Takara) reagent and gene-specific primers in an LC480 PCR machine (Roche). PCR conditions were as follows: initial denaturation: 95 °C for 5 min, 45 cycles of amplification: 95 °C for 10 s, 60 °C for 10 s, 72 °C for 10 s, and melting curve analysis: 65 °C to 97 °C at 0.1 °C/s. Expression of target genes was normalized to internal reference genes (hamster γ-actin or GAPDH) and the comparative Ct (2-ΔΔCt) method utilized to calculate the cytokine expression profile.

### Histopathology and immunostaining of viral antigens

Organs were fixed in 10% PBS buffered formalin and processed into paraffin-embedded blocks. Tissue sections were stained with haematoxylin and eosin (H&E) and examined by light microscopy as in a previous study^47^. For staining of viral antigens in lung tissue, indirect immunofluorescence assay was used^48^. Samples were deparaffinized and rehydrated. After blocking with normal goat serum for 30 min at room temperature, a rabbit anti–SARS-CoV2 nucleocapsid antibody^49^ (1:1000 dilution) was added and incubated at 4 °C overnight, followed by washing and incubation with a FITC-conjugated goat anti-rabbit IgG antibody (Abcam) (1:500 dilution) for 1 h at room temperature. Images were captured by confocal microscopy using a Carl Zeiss LSM 700.

### T cell response analysis

Nine 6–8-week-old BALB/c mice were transduced with Ad5-hACE2 virus (4 × 10^8^ pfu)^50^. After 5 days, the mice were immunized with Ca-DelMut (1 × 10^5^ pfu) (*n* = 5) or mock immunized with PBS (*n* = 4). After 4 weeks, lung and spleen tissues were collected. Splenocytes were isolated and homogenized through a cell strainer (BD), then resuspended in RPMI medium (10% fetal bovine serum (FBS) and penicillin/streptomycin). Lung tissue was chopped and digested in RPMI solution containing collagenase II (1 mg/ml) (Sigma) and DNase (10 mg /ml) (Roche) for 1 h at 37 °C. Red blood cells were lysed by addition of Lysing solution (BD). After three washes with RPMI, cells were counted and resuspended in RPMI. Cells were stimulated in RPMI solution containing 1 mg/ml of either spike or NP peptide pool (sets of 15-mers) overlapping by 11 amino acids (spanning the whole protein) or no peptide control. After 1 h, brefeldin A (BFA) was added and cells were incubated overnight at 37 °C. Cells were then washed with FACS buffer (2% FBS in PBS) and stained with anti-CD8α Pacific Blue (PB), anti-CD4 Allophycocyanin (APC), and Zombie (Biolegend) for 30 min at 4 °C. Cells were washed and fixed with Perm/Wash buffer (BD), and then stained with anti-IFN-γ-PE, anti-TNF-α-FITC, and anti-IL2- R-phycoerythrin/cy7 (PE/Cy7) (Biolegend) overnight at 4 °C. Cells were then washed twice with FACS buffer and resuspended in FACS buffer. Sample data were acquired using a BD FACSAria III cell sorter and the generated data were analyzed with FlowJo V9 (Supplementary Fig. 9). Cytokine production was calculated by subtracting the background of the no peptide control.

### Generation of pseudovirus and virus entry assay

293T cells were co-transfected with human immunodeficiency virus type 1 pNL4-3-Luc^+^Env^−^Vpr^−^ plasmid and WT or different mutant spike protein expression plasmids using TransLT1 (Mirus). After 48 h, viral supernatant was collected, aliquoted, and stored at −80 °C. Virus titers were quantified by measuring/p24 antigen levels using the HIV p24 ELISA kit (Sino Biological). To study virus entry, equal amounts of pseudovirus were added to 293T-hACE2 stable cell cultures. After 48 h, cells were lysed and luciferase activity measured using a luciferase assay kit (Promega).

### Statistical analysis

Statistical analysis was carried out using GraphPad Prism software (GraphPad Software). Data were presented as mean ± SD of at least 3 replicates, unless otherwise indicated. Statistical significance was analyzed by either Student’s *t*-test, one-way analysis of variance (ANOVA) with Tukey post-test, or two-way ANOVA with Bonferroni post-test. For all tests: ^****^p < 0.0001, ^***^p < 0.001, ^**^p < 0.01, ^*^p < 0.05, NS, not significant.

### Reporting summary

Further information on research design is available in the Nature Research Reporting Summary linked to this article.

## Supplementary information

Supplementary information.

Reporting summary.

## Data Availability

The sequences used in Fig. 1 are available via GenBank (accession code MT862537). Source data are provided with this paper.

## Source data

Source Data.
