# Supplementary material for: Characterization of an attenuated SARS-CoV-2 variant with a deletion at the S1/S2 junction of the spike protein
Source: Nat Commun. 2021 May 13;12:2790. doi: 10.1038/s41467-021-23166-0 (PMC8119425; doi:10.1038/s41467-021-23166-0)
Supplement: Supplementary file 1 — Supplementary information. [file 41467_2021_23166_MOESM1_ESM.pdf]

Characterization of an attenuated SARS-CoV-2 variant with a deletion at the S1/S2 junction of the spike protein

Supplementary Table 1*

Deletion and mutations in Ca-DelMut virus genome compared to Wuhan-Hu-1

**Nucleotide coordinate**	**Wuhan-Hu-1**	**Ca-DelMut**			
		**Nucleotide**	**Amino acid**	**Protein**	**ORF**
1663	C	**T**	**No change**	NSP2	ORF1a
4455	C	**T**	**A578V**	NSP3a	ORF1a
8782	C	**T**	**No change**	NSP4	ORF1a
21636	C	**T**	**P25L**	S	S
22661	G	**T**	**V367F**	S	S
23598-23627	No deletion	**30-base-pair deletion**	**10 aa deletion**	S	S
24034	C	**T**	**No change**	S	S
26303	T	**C**	**F20S**	E	E
26729	T	**C**	**No change**	M	M
28077	G	**C**	**V62L**	ORF8	ORF8
28144	T	**C**	**L84S**	ORF8	ORF8

*Details of deletion and mutations in the Ca-DelMut variant with reference to the Wuhan-Hu-1 SARS-CoV-2 strain [1]

Supplementary Table 2: List of primers used in this study

List of primers for sequencing of Ca-DelMut

List of primers for genome sequencing of SARS-CoV-2

Name	Sequence
HKUSZ2-1F	CAACCAACTTTCGATCTCTTGTAGATCTG
HKUSZ2-49F	AATCTGTGTGGCTGTCACTCGG
HKUSZ2-111F	ATTACTGTCGTTGACAGGACACGAG
HKUSZ2-156F	GGCTGCTTACGGTTTCGTCCG
HKUSZ2-501F	GCAGAACTCGAAGGCATTCAGTAC
HKUSZ2-967F	CTGAAAAGAGCTATGAATTGCAGACAC
HKUSZ2-1487F	CAAGTGTGCCTATTGGGTTCCAC
HKUSZ2-2001F	TTGGCTACTAACAATCTAGTTGTAATGG
HKUSZ2-2501F	TGTCTTGAAAACTGGTGATTTACAACC
HKUSZ2-3001F	CTTTCTACCCTCCAGATGAGGATG
HKUSZ2-3478F	CCATGCAAGTTGAATCTGATGATTAC
HKUSZ2-4011F	AATGGCAATCTTCATCCAGATTCTG
HKUSZ2-4508F	CACCAGTAAAACAACTGTAGCGTCAC
HKUSZ2-5001F	TATGGACAACAGTTTGGTCCAAC
HKUSZ2-5477F	GAACGTGGTGTGTAAAACTTGTGG
HKUSZ2-5979F	CCAAACCAACCATATCCAAACG
HKUSZ2-6516F	CTAATGGCTGCTTATGTAGACAATTCTAG
HKUSZ2-7007F	CATGCCTTCTTACTGTACTGGTTACAGAG
HKUSZ2-7504F	CAACTATTGTTAATGGTGTTAGAAGGTCC
HKUSZ2-7999F	AAATGTTTGATGCTTACGTTAATACG
HKUSZ2-8477F	ACAAGTTGTTAATGTTGTAACAACAAAGATAG
HKUSZ2-9001F	AAGATGCTTCTGGTAAGCCAGTACC
HKUSZ2-9478F	CTTTACTATTCCTTATGTCATTCACTGTACTC
HKUSZ2-9981F	TACCAACCACCACAAACCTCTATCAC
HKUSZ2-10515F	ATGGAATTACCAACTGGAGTTCATGC
HKUSZ2-10994F	TTTGACTTCACTTTTAGTTTTAGTCCAG
HKUSZ2-11498F	CAGAGGTATTGTTTTTATGTGTGTTGAG
HKUSZ2-11993F	CATGCAGGGTGCTGTAGACATAAAC
HKUSZ2-12508F	CATCAGCATTGTGGGAAATCCAAC
HKUSZ2-12996F	GGTAATGCAACAGAAGTGCCTGC
HKUSZ2-13501F	ATACAGGGCTTTTGACATCTACAATG
HKUSZ2-14001F	ATGCTGGTATTGTTGGTGTACTGAC
HKUSZ2-14506F	TAGTTTTAAGGAATTACTTGTGTATGCTGC
HKUSZ2-15000F	CAAAACGTAATGTCATCCCTACTATAACTC
HKUSZ2-15498F	GTCAAGCTGTCACGGCCAATG
HKUSZ2-16002F	ATGCTTACCCACTTACTAAACATCCTAATC
HKUSZ2-16499F	GGTAGCGATAATGTTACTGACTTTAATG
HKUSZ2-17003F	GCAAATTATCAAAAGGTTGGTATGC
HKUSZ2-17509F	CATGTTCCTCGGAACTTGTCGG

HKUSZ2-18000F	CTTTACAAGCTGAAAATGTAACAGGAC
HKUSZ2-18473F	GGACTTCCTTGGAATGTAGTGCG
HKUSZ2-18991F	TCTTCACGACATTGGTAACCCTAAAG
HKUSZ2-19461F	GACATCATGCTAATGAGTACAGATTGTATC
HKUSZ2-19999F	CCGTAATGGTGTTCTTATTACAGAAGG
HKUSZ2-20507F	AAATCCCAAGATTTATCTGTAGTTTCTAAGG
HKUSZ2-20998F	GGATCTCATTATTAGTGATATGTACGACCC
HKUSZ2-21431F	GATATGATTTTATCTCTTCTTAGTAAAGGTAGAC
HKUSZ2-21992F	AAGTGAGTTCAGAGTTTATTCTAGTGCG
HKUSZ2-22486F	GAGTCCAACCAACAGAATCTATTGTTAG
HKUSZ2-22961F	CACACCTTGTAATGGTGTTGAAGG
HKUSZ2-23511F	GAGTGTGACATACCCATTGGTGC
HKUSZ2-24001F	ACAAAGTGACACTTGCAGATGCTG
HKUSZ2-24492F	GAGGCTGAAGTGCAAATTGATAGG
HKUSZ2-25001F	GAATCATACATCACCAGATGTTGATTTAG
HKUSZ2-25493F	TGGCTTATTGTTGGCGTTGCAC
HKUSZ2-25972F	CAGTTACTTCACTTCAGACTATTACCAGC
HKUSZ2-26501F	CCAACGGTACTATTACCGTTGAAG
HKUSZ2-26998F	GTTGCTACATCACGAACGCTTTC
HKUSZ2-27492F	CACCATTTCATCCTCTAGCTGATAAC
HKUSZ2-28019F	AATCAGCACCTTTAATTGAATTGTGC
HKUSZ2-28494F	GGCTACTACCGAAGAGCTACCAG
HKUSZ2-28995F	GCTGAGGCTTCTAAGAAGCCTCG
HKUSZ2-29503F	CTCATGCAGACCACACAAGGCAG
HKUSZ2-670R	CCTAAGTCAAATGACTTTAGATCGGC
HKUSZ2-1158R	GTGACGCAACTGGATAGACAGATCG
HKUSZ2-1698R	CCACAAAAGCACTTGTGGAAGCAG
HKUSZ2-2167R	CCGTCTCTAAGAAACTCTACACCTTCC
HKUSZ2-2663R	GTTTGTTACCATCATATTAGGTGCAAG
HKUSZ2-3152R	TTCTTCAGGTTGAAGAGCAGCAGAAG
HKUSZ2-3673R	GGTGCAAGTAGAACTTCGTGCTG
HKUSZ2-4173R	CTAGCATTTCAGTAGTGCCACCAG
HKUSZ2-4669R	GAAGAAACAGAAACTGTAGCTGGCAC
HKUSZ2-5049R	TAGTAACATCAGCTCCATCCAAATAAG
HKUSZ2-5674R	TACTGAGCAGGTGGTGCTGACATC
HKUSZ2-6143R	AATAGCCACCACATCACCATTTAAGTC
HKUSZ2-6685R	CTAACAACTTTGTTAAGAAAAGGCTTAGC
HKUSZ2-7162R	GTAATTTGTATAGTTTCTAAAGAAGGATAGGTGTC
HKUSZ2-7663R	AACTGTAGTGACAAGTCTCTCGCAAC
HKUSZ2-8163R	AATCAACAAACCCTTGCCGAGC
HKUSZ2-8667R	TAGCCTTGTATCCTATGATTTCACTTG
HKUSZ2-9168R	AAGTTGTTACCACTCTAACAGAACCTTC
HKUSZ2-9655R	GTTATCCAGAAAGGTACTAAAGGTGTGAAC
HKUSZ2-10170R	GCATGTCTTCAGAGGTGCAGATC
HKUSZ2-10653R	CAGCGTACAACCAAGCTAAAACATTAAC

HKUSZ2-11179R	TAAGCTACAGTGGCAAGAGAAGGTAAC
HKUSZ2-11658R	CAAGAGTCAGTCTAAAGTAGCGGTTG
HKUSZ2-12162R	GAACAACTTCAGAATCACCATTAGC
HKUSZ2-12668R	ACTAAGCTCATTATTCTGTAATTTGACAGC
HKUSZ2-13159R	GTTATTGCCTGACCAGTACCAGTGTG
HKUSZ2-13658R	CTTCATGTTGGTAGTTAGAGAAAGTGTG
HKUSZ2-14166R	GCAGTTAAAGCCCTGGTCAAGG
HKUSZ2-14669R	CATAGAAGTCTTTGTTAAAATTACCGGG
HKUSZ2-15164R	TAGCTCCTCTAGTGGCGGCTATTG
HKUSZ2-15669R	GAGAAATGTTTACGCAAATATGCG
HKUSZ2-16164R	TCAGGTTCCCAATACCTTGAAGTG
HKUSZ2-16670R	CACGTACAGTAGCAATACCATAAGACAG
HKUSZ2-17164R	CTTCTCACATAGTGCATCAACAGC
HKUSZ2-17663R	CAGATGAAACATCATGCGTGATAACAC
HKUSZ2-18069R	GTAGGATGTAACCCAGTGATTACCTTAC
HKUSZ2-18655R	GCATGTGGCACGTCTATCACATAG
HKUSZ2-19058R	AGAACTTCCATTCTACATCAGCTTGAG
HKUSZ2-19662R	GAAACTGGTACTTCACCCTGTTGTCC
HKUSZ2-20156R	GTAATTGTTGGACAACACCATCAAC
HKUSZ2-20665R	ATTAGGCATAGCAACACCCGGTTG
HKUSZ2-21165R	AGATCAGCATTCCAAGAATGTTCTG
HKUSZ2-21668R	TGAGGATCTGAAAACTTTGTCAGGG
HKUSZ2-22186R	CCCTGAGGGAGATCACGCAC
HKUSZ2-22631R	GACAGAATAATCAGCAACACAGTTGC
HKUSZ2-23170R	GTTAAACCATTGAAGTTGAAATTGACAC
HKUSZ2-23569R	GAATTAGTCTGAGTCTGATAACTAGCGC
HKUSZ2-24166R	GCTAACAGTGCAGAAGTGTATTGAGC
HKUSZ2-24683R	GGACATAAGATGATAGCCCTTTCC
HKUSZ2-25158R	TATACTGCTCATACTTTCCAAGTTCTTG
HKUSZ2-25667R	CTTCAAGGCCAGCAGCAACGAG
HKUSZ2-26164R	CGTCGGTTCATCATAAATTGGTTCC
HKUSZ2-26665R	GCCATAACAGCCAGAGGAAAATTAAC
HKUSZ2-27140R	ATATTGTCACTGCTACTGGAATGGTC
HKUSZ2-27650R	AAAGTTCTTGAACTTCCTCTTGTCTG
HKUSZ2-28170R	CGAACAACGCACTACAAGACTACCC
HKUSZ2-28665R	GTGTATTCAAGGCTCCCTCAGTTGC
HKUSZ2-29164R	GCAAATTGTGCAATTTGCGGC
HKUSZ2-29660R	CACACTGATTAAAGATTGCTATGTGAG
HKUSZ2-29719R	CGTACTCCGCGTGGCCTCG
HKUSZ2-29767R	TCTTCCATATAGGCAGCTCTCCCTAG
HKUSZ2-29838R	GTCATTCTCCTAAGAAGCTATTAAAATCACATG

List of primers for quantification of cytokine expression in hamster

	Forward	Reverse
Hamster IFN-γ	TGTTGCTCTGCCTCACTCAGG	AAGACGAGGTCCCCTCCATTC
Hamster IL-4	ACAGAAAAAGGGACACCATGCA	GAAGCCCTGCAGATGAGGTCT
Hamster IL-6	AGACAAAGCCAGAGTCATT	TCGGTATGCTAAGGCACAG
Hamster IL-10	GGTTGCCAAACCTTATCAGAAATG	TTCACCTGTTCCACAGCCTTG
Hamster IL-13	AAATGGCGGGTTCTGTGC	AATATCCTCTGGGTCTTGTAGATGG
Hamster TNF-α	TGAGCCATCGTGCCAATG	AGCCCGTCTGCTGGTATCAC
Hamster IL-21	GGACAGTGGCCCATA AAACAAG	TTCAACACTGTCTATAAGATGACGAAGTC
Hamster TGFβ1	GGCTACCACGCCAACTTCTG	GAGGGCAAGGACCTTACTGTACTG
Hamster CCL17	GTGCTGCCTGGAGATCTTCA	TGGCATCCCTGGGACACT
Hamster CCL22	TGGTGCCAA CGTGGAAGAC	GAAGAACTCCTTCACTACGCGC
Hamster CCR4	GCTTGGTCACGTGGTCAGTG	GTGGTTGCGCTCCGT GTAG
Hamster FOXP3	GGTCTTCGAGGAGCCAGAAGA	GCCTTGCCCTTCTCATCCA
Hamster IL-12p40	AATGCGAGGCAG CAAATTACTC	CTGCTCTTGACGTTGAACTTCAAG
Hamster γ-actin	ACAGAGAGAAGATGACGCAGATAATG	GCCTGA ATGGCCACGTACA
Hamster GAPDH	GGTTGCCAAACCTTATCAGAAATG	TTCACCTGTTCCACAGCCTTG

Supplementary Figure 1

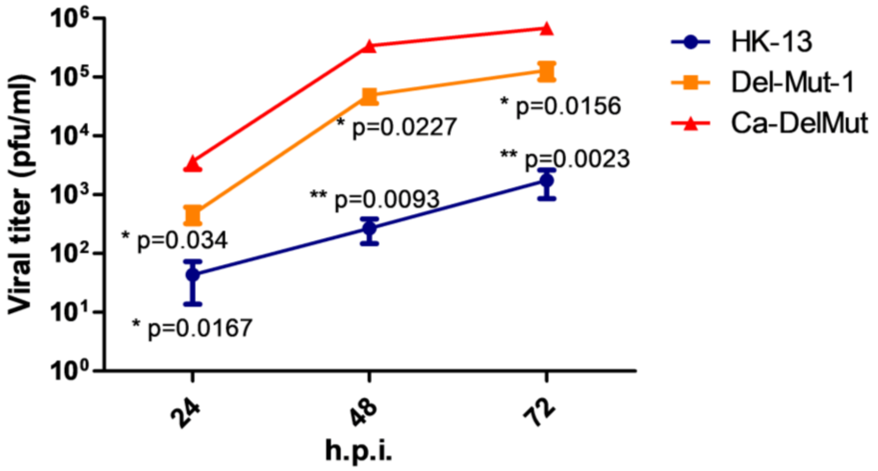

Supplementary Figure 1. Ca-DelMut propagates faster than WT virus at 30°C *in vitro*. Vero E6 cells were infected with Ca-DelMut and other viruses at 0.01 MOI and incubated at 30°C. At the indicated time points, supernatants were collected and virus titer determined by plaque assay. Statistical comparisons between means were performed by one-way ANOVA: ** $p<0.01$, * $p<0.05$. Error bars represent mean ± SD (n=3). h.p.i.: hours post infection.

Supplementary Figure 2

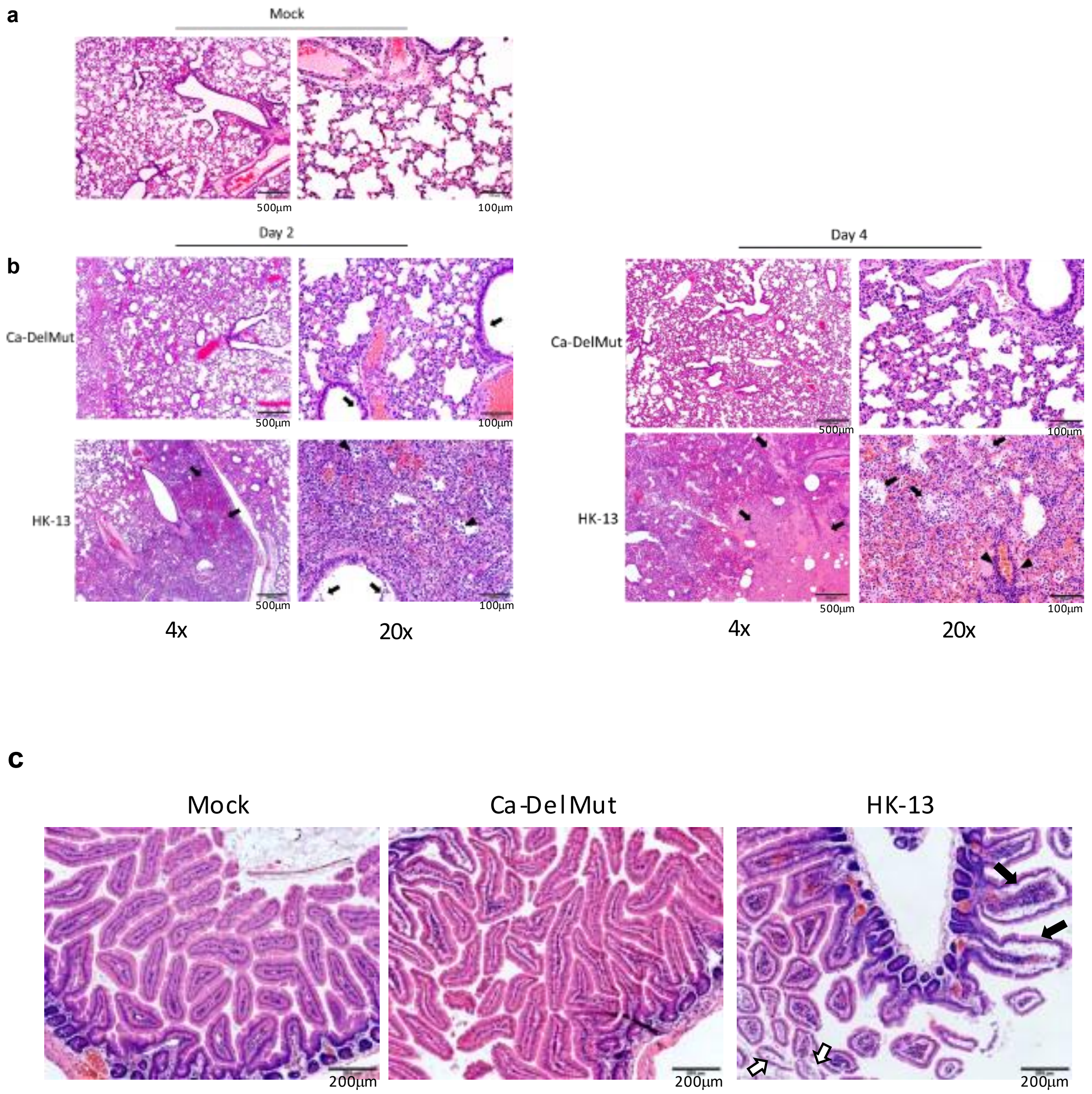

Supplementary Figure 2. Infection with Ca-DelMut causes only mild pathological changes. Hamsters were infected with $1x10^3$ pfu of either Ca-DelMut or HK-13 (WT), or mock infected. At days 2 and 4 post-infection, lungs and small intestine were collected and fixed in 10% formalin, and then processed into paraffin blocks and sections H&E stained. Images were representatives of three independent experiments.

(A) The top panel shows normal lung structures in the lungs of mock-infected control hamsters at 4x (left) and 20x (right) magnification.

(B). At day 2 after infection with Ca-DelMut, lung tissues showed only mild regional alveolar septal infiltration and blood vessel congestion. No obvious bronchiolar epithelium desquamation or luminal debris (arrows), and no alveolar space infiltration or exudation were observed. At day 4, no deleterious progression of histopathology was observed. For WT virus at day 2 post-infection, the low magnification image (left) showed regional lung consolidation and focal pulmonary hemorrhage (arrows). The higher magnification image (right) showed

massive alveolar space infiltration (arrowheads) and hemorrhage, with a little bronchiolar luminal cell debris (arrows) visible. At day 4 post WT-infection, the low magnification image (left) showed intensive alveolar exudation, infiltration and hemorrhage resulting in pulmonary consolidation (arrows), while the higher magnification image (right) showed intensive protein rich exudates filling the alveolar space (arrows), in addition to massive infiltration and alveolar hemorrhage; a blood vessel shows moderate infiltration (arrowheads).

(C) Representative histological images of hamster small intestines. The left-most image shows mock-infected control hamster small intestinal villi with normal structure. At day 4 post infection with Ca-DelMut, no apparent histopathological changes were detected in the small intestine (middle). For WT virus infection, small intestinal lamina propria blood vessel congestion, infiltration and edema resulting in swelling of the villi (solid arrows) and enterocyte desquamation (open arrows) were observed.

Supplementary Figure 3

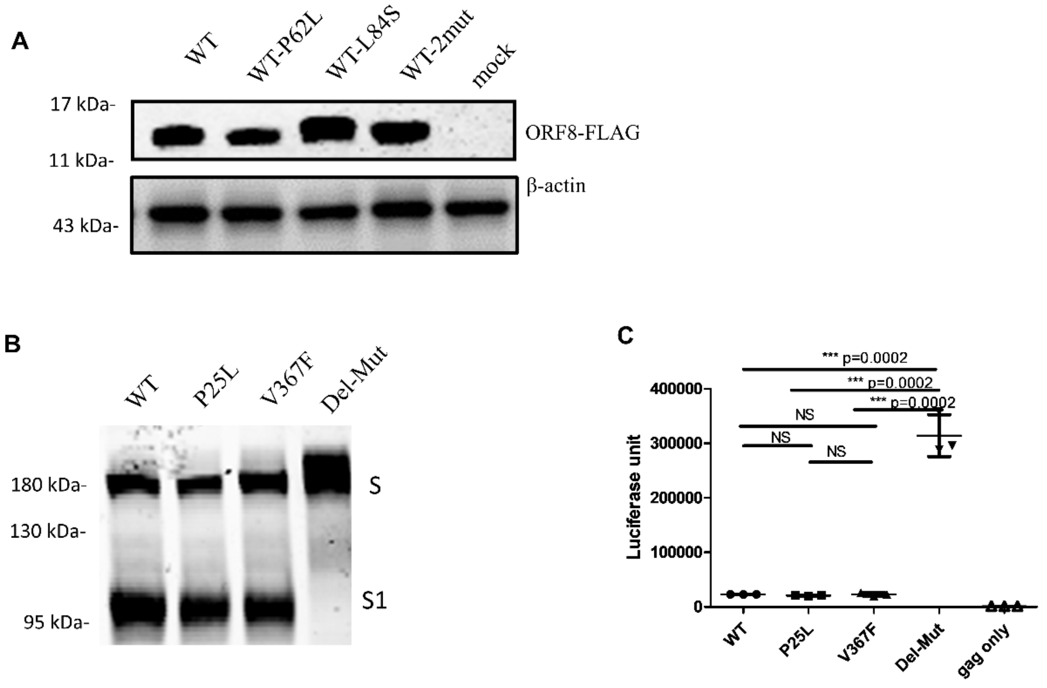

Supplementary Figure 3. Effect of different mutations on viral protein properties in Ca-DelMut variant. (A) 293T cells were transfected with C terminal Flag-tagged wild type or mutant ORF8 (P62L (Fig 1 and Supp Table 1 both say V62L), L84S, or both V62L and L84S (WT-2mut): Figure 1 & Supplementary Table 1) expressing plasmids. Cell lysates were collected at 48 hours post-transfection and ORF8 expression detected by western blotting using anti-Flag antibody. β-actin was used as a loading control. (B) 293T cells were transfected with plasmids for expression of wild type or mutant spike proteins (P25L, V367F or 30bp deletion at S1/S2 junction (Del-Mut): Figure 1 & Supplementary Table 1). Cell lysates were collected at 48 hours post-transfection. Full length spike and cleaved S1 subunit were analyzed by western blot using a polyclonal anti-spike RBD antibody. (C) Effect of spike mutations on pseudovirus entry in 293T cells. Pseudoviruses containing WT or mutant spike genes (P25L, V367F or P25L/V367F dual mutations, or Del-Mut) were prepared and quantified as indicated in Methods. Equal amounts of pseudovirus were added to 293T-hACE2 stable cell cultures to estimate virus entry. At 48 hours post-inoculation, cells were lysed and luciferase activity measured using a luciferase assay kit. Pseudovirus without expressing spike protein (gag only) is used as a control. Statistical comparisons between means were performed by one-way ANOVA: *** $p<0.001$, NS: not significant. Error bar represents mean ± SD (n=3).

Supplementary Figure 4

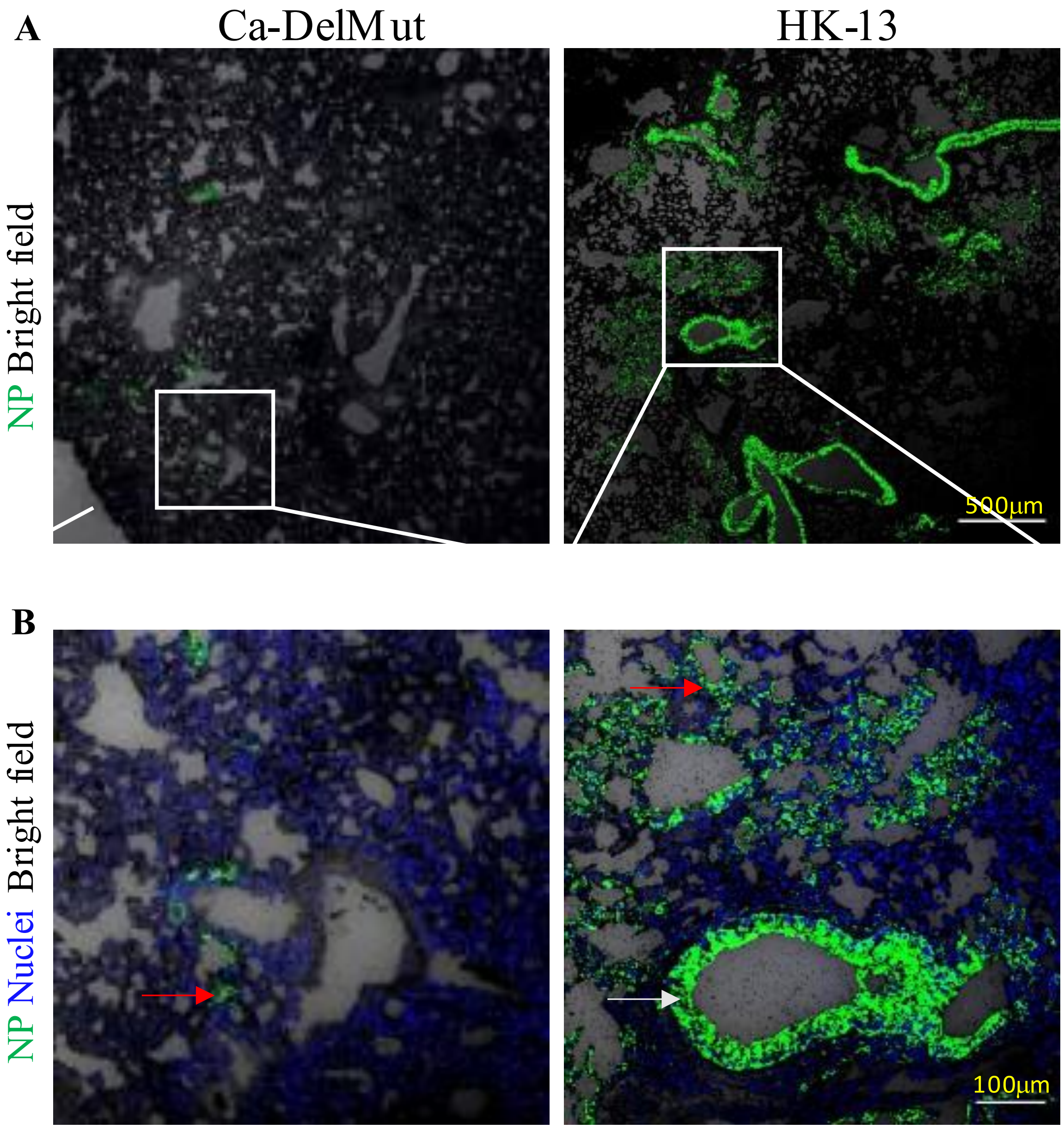

Supplementary Figure 4. Tissue tropism of Ca-DelMut and WT HK-13 virus in the lungs of hamsters. Sections are representative images of viral antigen (NP)-positive signals detected in the epithelial cells of bronchi (white arrow) and pneumocytes (red arrow) in wild-type SARS-CoV-2 infected lungs (right panels), and pneumocytes (red arrow) in Ca-DelMut infected lungs (left panels). Lung tissue samples were stained for SARS-CoV-2 nucleocapsid protein (NP: FITC). DAPI staining indicates nuclei. The upper panel shows a low magnification overview (X50, scale bar: 500um). The bottom panel shows a higher magnification of the boxed area in the upper panel (X200, scale bar: 100um). Images were representatives of three independent experiments.

Supplementary Figure 5

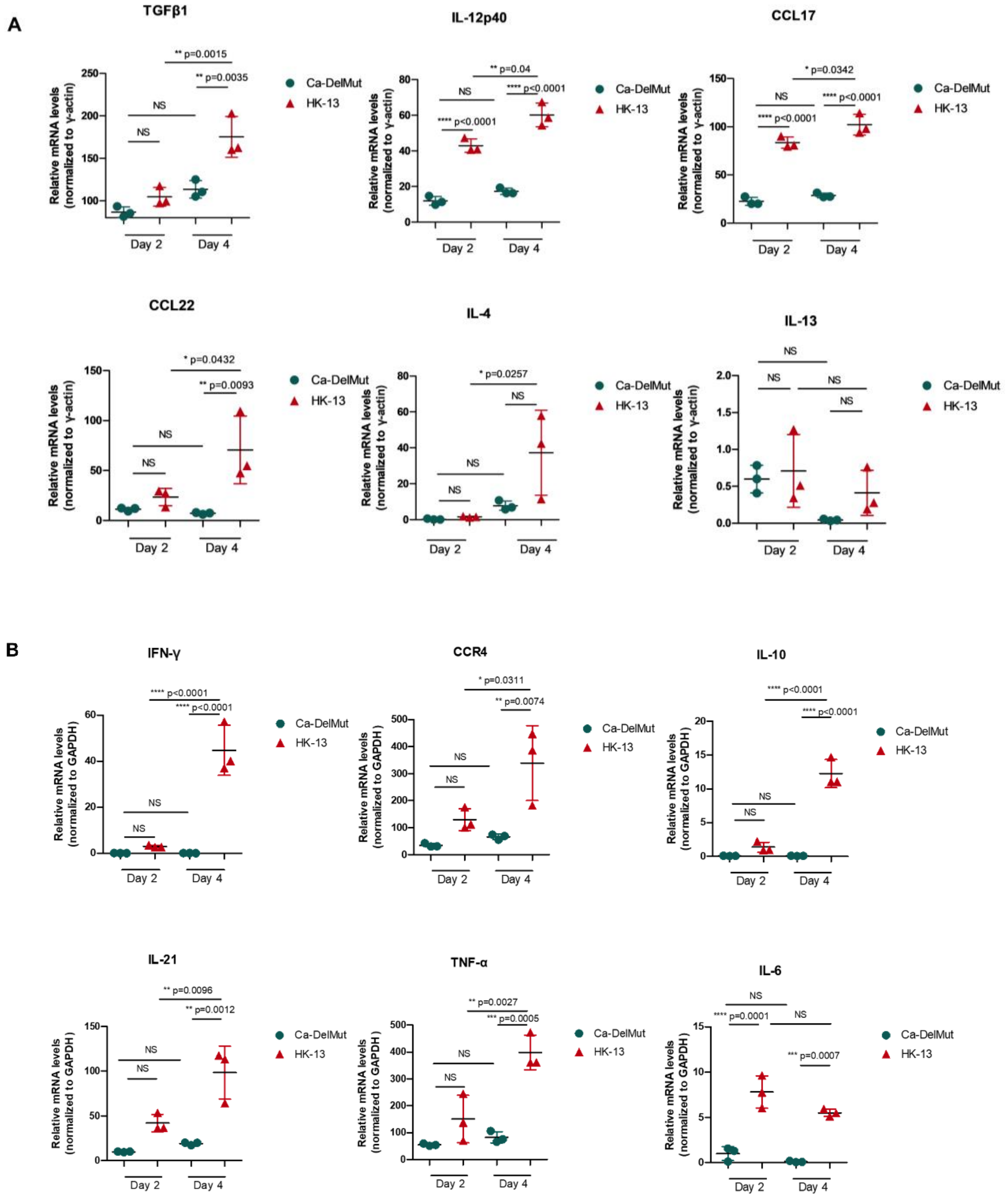

Supplementary Figure 5. Ca-DelMut does not induce elevated levels of proinflammatory cytokines in hamsters. Hamsters were infected intranasally with $1x10^3$ pfu of either Ca-DelMut or WT HK-13 virus. At days 2 and 4, RNA was extracted from lung and nasal turbinate tissues using RNAsol RT procedures. cDNA was synthesized using oligo dT primers. Expression of different proinflammatory cytokines was examined by qPCR, normalized to the internal reference genes hamster γ-actin (A) or GAPDH (B), and the comparative Ct (2-ΔΔCt) method was utilized to calculate the cytokine expression profile. Error bar represents mean ± SD (n=3). Statistical comparisons between means were

performed by Student's t-test (two-tailed): **** $p<0.0001$, *** $p<0.001$, ** $p<0.01$, * $p<0.05$, NS: not significant.

Supplementary Figure 6

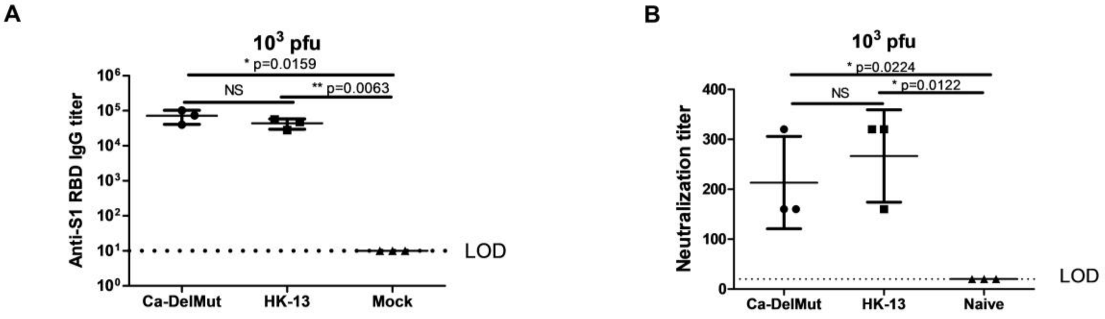

Supplementary Figure 6. Lower dose (10^3 pfu) Ca-DelMut immunization is able to induce a strong humoral response in hamsters. Hamsters were infected intranasally with $1x10^3$ pfu of either Ca-DelMut (n=3)or HK-13 virus (n=3), or mock immunized (n=3). At day 21, blood was collected from hamsters and tested for anti-S1 RBD IgG titers (a) and neutralization activity against HK-13 virus (b). Statistical comparisons between means were performed by one-way ANOVA: * $p<0.05$, NS: not significant. Error bars represent mean ± SD (n=3). LOD: level of detection.

Supplementary Figure 7

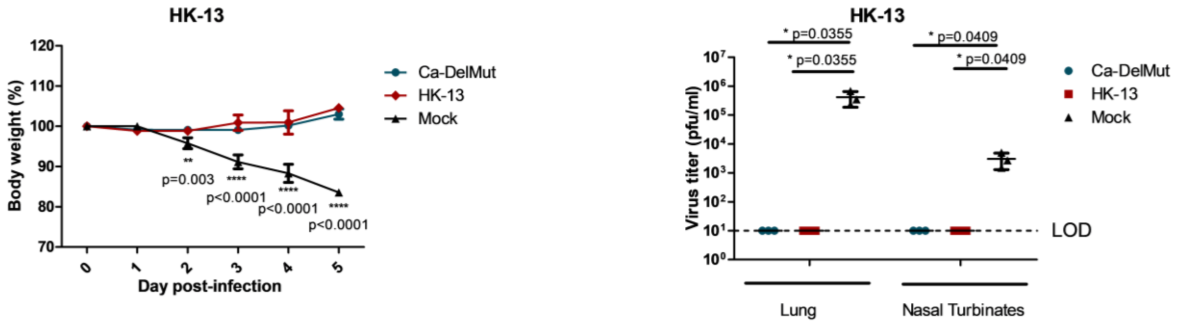

Supplementary Figure 7. Lower dose Ca-DelMut immunization still provides complete protection against WT HK-13 virus. Hamsters were infected intranasally with 1×10^3 pfu of either Ca-DelMut (n=3) or HK-13 virus (n=3), or mock immunized (n=3). At day 28 after immunization, hamsters were challenged with 1×10^3 pfu of HK-13 virus. Body weight change and disease symptoms were monitored for 5 days. At day 5 post-infection, lungs and nasal turbinate tissues were collected for virus titration. Statistical comparisons between means were performed by two-way ANOVA (body weight) or one-way ANOVA (virus titer): * $p<0.05$. Error bars represent mean ± SD (n=3).

Supplementary Figure 8

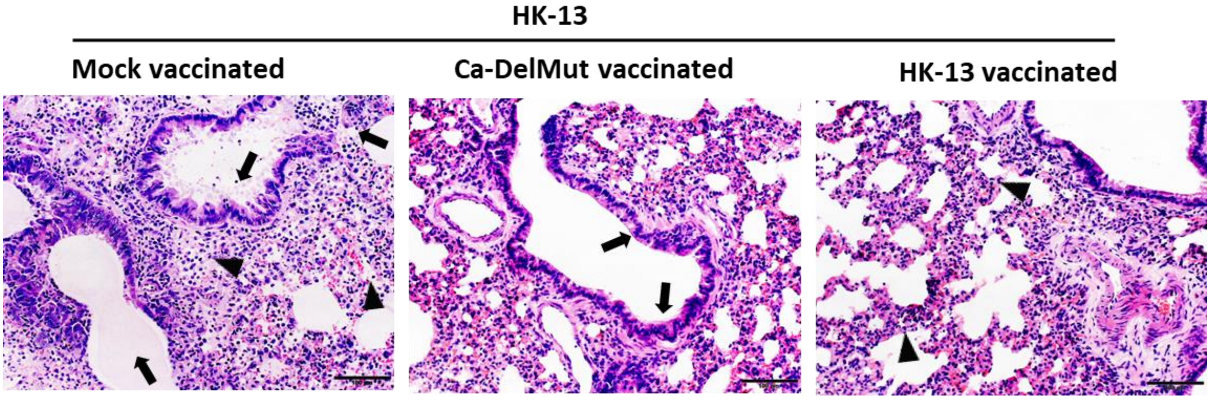

Supplementary Figure 8. A low inoculum of Ca-DelMut provides protection against infection with wild type SARS-CoV-2. Hamsters were infected intranasally with $1x10^3$ pfu of either Ca-DelMut, the HK-13 strain or mock immunized. At day 28 after immunization, hamsters were challenged with $1x10^3$ pfu of HK-13 virus. At day 5 post infection, lungs were collected for histopathological study. Lungs were fixed in 10% formalin, and then processed into paraffin blocks and H&E staining performed. Mock vaccinated hamster lung showed bronchiolar epithelial cell death and luminal secretion mixed with cell debris (arrows) and diffuse alveolar infiltration and exudation (arrowheads), while Ca-DelMut immunized lung showed no apparent bronchiolar epithelium cell death (arrows) and alveoli showed regional septal infiltration. In HK-13 immunized hamster lung, no apparent histopathology other than alveolar wall thickening (arrowheads) was observed following re-challenge. Indicate size of scale bars. Images were representatives of three independent experiments.

Supplementary Figure 9

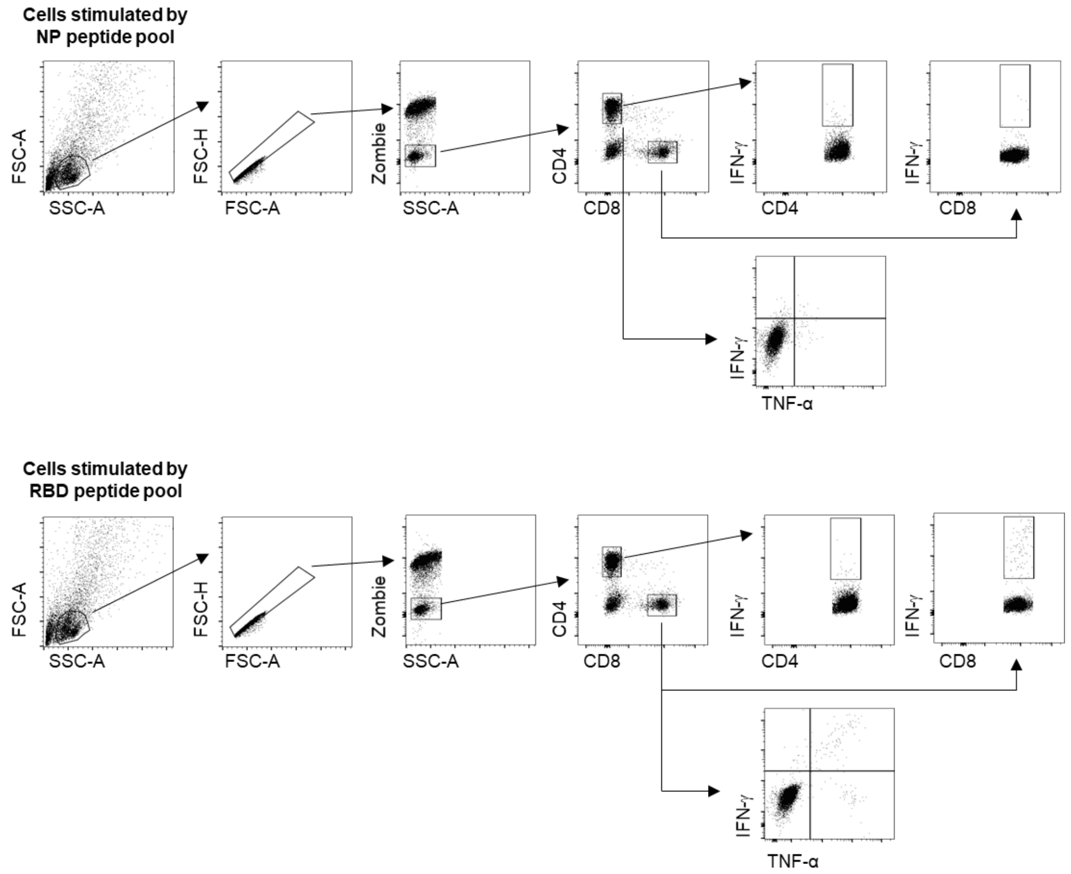

Supplementary Figure 9. Flow cytometry gating strategy: The cells were gated with FSC-H vs FSC-A for single cells, and then gated with zombie vs SSC-A for live cells, and then gated with CD4+ vs CD8+ for the two T cell subsets, and then gated with IFN-γ vs CD4+ or CD8+. For IFN-γ and TNF-α double positive cells, CD4+ or CD8+ cells were gated with IFN-γ and TNF-α.

Supplementary References

1. Wu, F., et al., *A new coronavirus associated with human respiratory disease in China.* Nature, 2020. **579**(7798): p. 265-269.
