# Supplementary material for: Characterization of an attenuated SARS-CoV-2 variant with a deletion at the S1/S2 junction of the spike protein
Source: Nat Commun. 2021 May 13;12:2790. doi: 10.1038/s41467-021-23166-0 (PMC8119425; doi:10.1038/s41467-021-23166-0)
Supplement: Supplementary file 2 — Reporting summary. [file 41467_2021_23166_MOESM2_ESM.pdf]

nature research

Corresponding author(s): Honglin Chen

Last updated by author(s): Mar 1, 2021

Reporting Summary

Nature Research wishes to improve the reproducibility of the work that we publish. This form provides structure for consistency and transparency in reporting. For further information on Nature Research policies, see our Editorial Policies and the Editorial Policy Checklist.

Statistics

For all statistical analyses, confirm that the following items are present in the figure legend, table legend, main text, or Methods section.

n/a	Confirmed	
☐	☒	The exact sample size (*n*) for each experimental group/condition, given as a discrete number and unit of measurement
☐	☒	A statement on whether measurements were taken from distinct samples or whether the same sample was measured repeatedly
☐	☒	The statistical test(s) used AND whether they are one- or two-sided<br>*Only common tests should be described solely by name; describe more complex techniques in the Methods section.*
☒	☐	A description of all covariates tested
☒	☐	A description of any assumptions or corrections, such as tests of normality and adjustment for multiple comparisons
☐	☒	A full description of the statistical parameters including central tendency (e.g. means) or other basic estimates (e.g. regression coefficient) AND variation (e.g. standard deviation) or associated estimates of uncertainty (e.g. confidence intervals)
☐	☒	For null hypothesis testing, the test statistic (e.g. *F*, *t*, *r*) with confidence intervals, effect sizes, degrees of freedom and *P* value noted<br>*Give P values as exact values whenever suitable.*
☒	☐	For Bayesian analysis, information on the choice of priors and Markov chain Monte Carlo settings
☒	☐	For hierarchical and complex designs, identification of the appropriate level for tests and full reporting of outcomes
☒	☐	Estimates of effect sizes (e.g. Cohen's *d*, Pearson's *r*), indicating how they were calculated

Our web collection on statistics for biologists contains articles on many of the points above.

Software and code

Policy information about availability of computer code

Data collection	qPCR data was done by Lightcycler 480 (Roche).<br>Confocal images were captured by LSM 700 (Carl Zeiss).<br>Flow cytometry was done by BD FACSAria III cell sorter (BD)<br>Absorbance data was measured by Fluostar Optima Microplate reader.<br>Western blot data was acquired by odyssey scanner (LICOR).
Data analysis	Statistical analysis was done by Graphpad prism Version 5.1.<br>Flow cytometry data was analyzed by FlowJo V9

For manuscripts utilizing custom algorithms or software that are central to the research but not yet described in published literature, software must be made available to editors and reviewers. We strongly encourage code deposition in a community repository (e.g. GitHub). See the Nature Research guidelines for submitting code & software for further information.

Data

Policy information about availability of data

All manuscripts must include a data availability statement. This statement should provide the following information, where applicable:

- Accession codes, unique identifiers, or web links for publicly available datasets
- A list of figures that have associated raw data
- A description of any restrictions on data availability

The data of this studies are available upon reasonable request.

Field-specific reporting

Please select the one below that is the best fit for your research. If you are not sure, read the appropriate sections before making your selection.

- [x] Life sciences
- [ ] Behavioural & social sciences
- [ ] Ecological, evolutionary & environmental sciences

For a reference copy of the document with all sections, see nature.com/documents/nr-reporting-summary-flat.pdf

Life sciences study design

All studies must disclose on these points even when the disclosure is negative.

Sample size	All experiments were repeated at least three times to give a n number of 3 or above. A n number equals to 3 is the the standard of biological experiments. No sample size calculation was performed. Sample size is chosen based on the standard of the corresponding field (PMID: 32215622).
Data exclusions	No data was excluded.
Replication	All experiments were repeated at least twice. Similar findings were obtained from all repeats.
Randomization	Randomization was applied to the grouping of the animals. Animals were randomly allocated to the groups.
Blinding	No blinding was done. Blinding was not relevant to the study because the results are quantitative and objective, and does not require a subjective judgment.

Reporting for specific materials, systems and methods

We require information from authors about some types of materials, experimental systems and methods used in many studies. Here, indicate whether each material, system or method listed is relevant to your study. If you are not sure if a list item applies to your research, read the appropriate section before selecting a response.

Materials & experimental systems

n/a	Involved in the study	
[ ]	[x]	Antibodies
[ ]	[x]	Eukaryotic cell lines
[x]	[ ]	Palaeontology and archaeology
[ ]	[x]	Animals and other organisms
[x]	[ ]	Human research participants
[x]	[ ]	Clinical data
[x]	[ ]	Dual use research of concern

Methods

n/a	Involved in the study	
[x]	[ ]	ChIP-seq
[ ]	[x]	Flow cytometry
[x]	[ ]	MRI-based neuroimaging

Antibodies

Antibodies used	Mouse anti-flag antibody (Sigma #F3165) 1:1000 for western blot.<br>Mouse anti-b-actin antibody (Sigma#A5441) 1: 5000 for western blot.<br>In house rabbit anti-SARS-CoV-2 Nucleocapsid antibody, 1:1000 for immunoflurosence.<br>In house rabbit anti-SARS-CoV-2 Spike RBD antibody, 1:1000 for western blot.<br>Rat Anti-mouse CD8a-PB (Biolegend #100516), 1: 200 for flow cytometry staining.<br>Rat Anti-mouse CD4-APC (Biolegend #100725),1: 200 for flow cytometry staining.<br>Rat Anti-mouse IFNg-PE (Biolegend #505808), 1: 100 for flow cytometry staining.<br>Rat Anti-mouse TNFa-FITC (Biolegend #506304), 1: 100 for flow cytometry staining.<br>Rat Anti-mouse IL2-PE/Cy7 (Biolegend #503832), 1: 100 for flow cytometry staining.
Validation	The commercial antibodies were validated by the manufacturers.<br>The in-house anti-SARS-CoV-2-N and anti-SARS-CoV-RBD immune serum were validated with ELISA, Western blots, and immunofluorescence staining in the previous publication (PMID: 32835326).

Eukaryotic cell lines

Policy information about cell lines

Cell line source(s)	Vero E6, Calu-3, 293T cells are from ATCC.

Authentication: The cell lines were authenticated by ATCC but were not authenticated by our group.

Mycoplasma contamination: All cell lines were tested negative for mycoplasma contamination.

Commonly misidentified lines (See ICLAC register): Nil

Animals and other organisms

Policy information about studies involving animals; ARRIVE guidelines recommended for reporting animal research

Laboratory animals: 6-8 weeks old Golden syrian hamsters and 6-8 weeks old female mouse (BALB/c) were used in this study.

Wild animals: Nil

Field-collected samples: Nil

Ethics oversight: All animal studies were approved by the Committee on the Use of Live Animals in Teaching and Research, Hong Kong University.

Note that full information on the approval of the study protocol must also be provided in the manuscript.

Flow Cytometry

Plots

Confirm that:

- [x] The axis labels state the marker and fluorochrome used (e.g. CD4-FITC).
- [x] The axis scales are clearly visible. Include numbers along axes only for bottom left plot of group (a 'group' is an analysis of identical markers).
- [x] All plots are contour plots with outliers or pseudocolor plots.
- [x] A numerical value for number of cells or percentage (with statistics) is provided.

Methodology

Sample preparation: Splenocytes were isolated and homogenized through cell strainer (BD), and resuspended in RPMI medium (10% FBS and P/S). Lung tissue was chopped and digested in RPMI solution with collagenase II (1 mg/ml) (Sigma) and DNase (10g /ml) (Roche) for 1h at 37 0C. Red blood cells were lysed by addition of Lysing solution (BD). After washes with RPMI, cells were counted and resuspended in RPMI.

Instrument: BD FACSAria III cell sorter

Software: Flowjo V9

Cell population abundance: No cell sorting was done.

Gating strategy: The cells were gated with FSC-H vs FSC-A for single cells, and then gated with zombie vs SSC-A for live cells, and then gated with CD4 vs CD8 for the two T cell subsets, and then gated with cytokines (IFNg, IL2, TNFa) vs CD4 or CD8.

- [x] Tick this box to confirm that a figure exemplifying the gating strategy is provided in the Supplementary Information.
